# Supplementary figures and images for: Using oxygen and hydrogen stable isotopes to track the migratory movement of Sharp-shinned Hawks (Accipiter striatus) along Western Flyways of North America
Source: PLoS One. 2020 Nov 17;15(11):e0226318. doi: 10.1371/journal.pone.0226318 (PMC7671529; doi:10.1371/journal.pone.0226318)

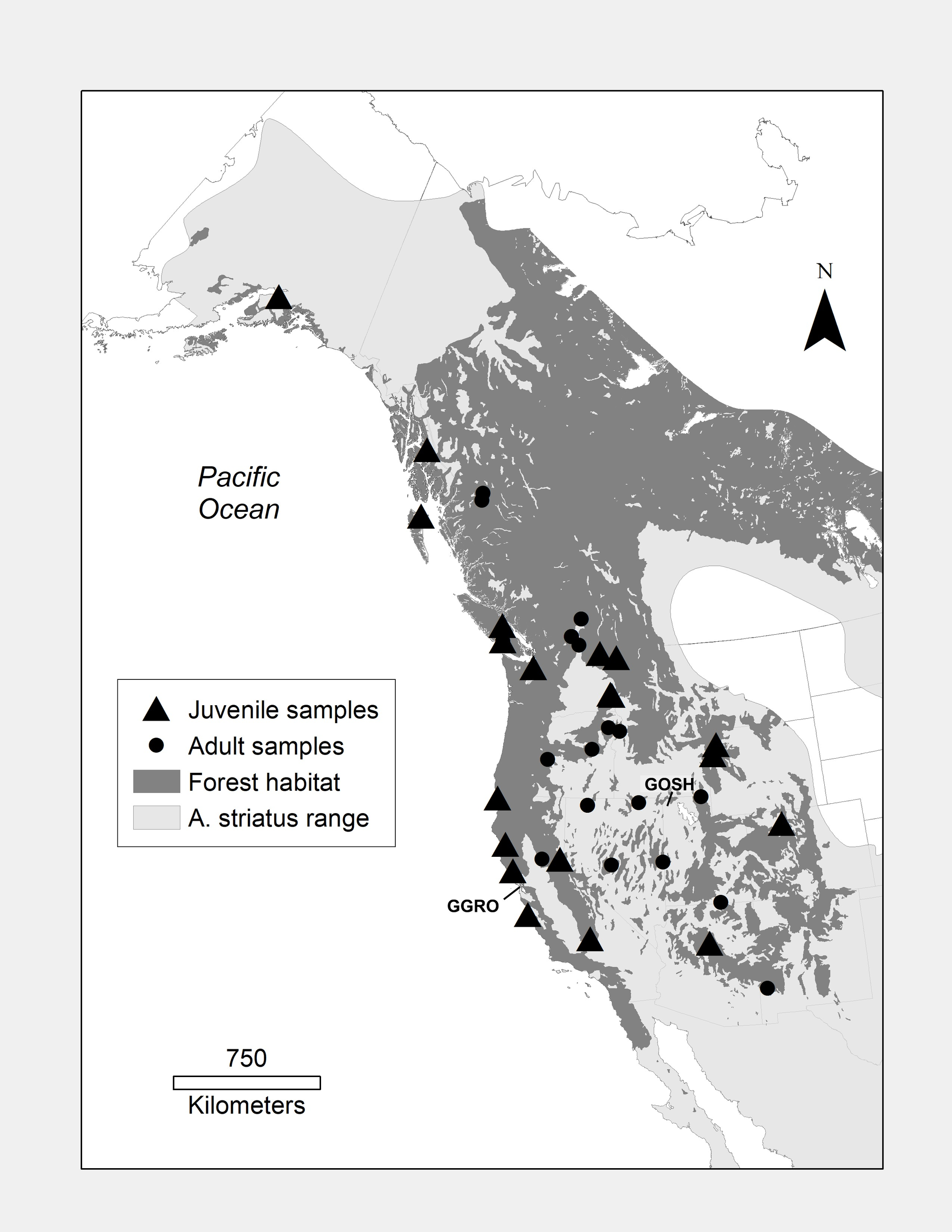

Supplement: S1 Fig — Sampling locations are shown in reference to the species known range in Western North America (light gray), and suitable breeding forest habitat (dark gray). Juveniles samples (n = 23) are shown as triangles, and adult samples (n = 25) are shown as circles. State and country boundaries are modified from public domain GIS files, US Census Bureau (2016) and Natural Earth (2020). Species range acquired from Birdlife International and NatureServe (2015), and data to create the GIS biome layer acquired from Brown, Bennan, and Unmack (2007). (TIF) [file pone.0226318.s001.tif]

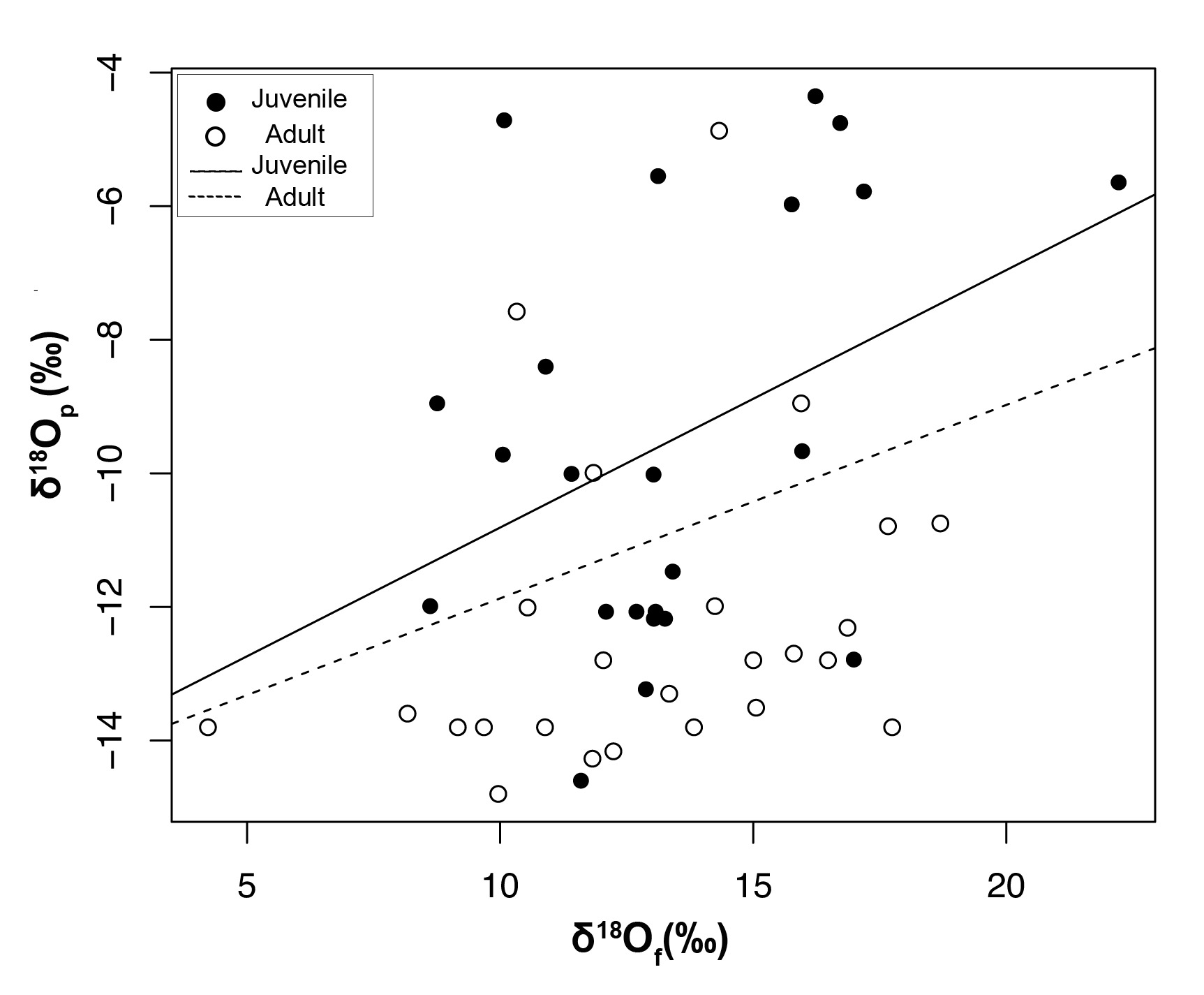

Supplement: S2 Fig — Stable oxygen (δ18OF ‰) isotopic composition of feathers for museum Sharp-shinned Hawk (Accipiter striatus) specimens of known natal/breeding origin and isoscape modeled isotopic composition of precipitation (δ18OP ‰) at the collection locations of (a) juvenile (black dots) birds as well as (b) adult birds (white dots) (n = 48). The linear regression for both juvenile and adult birds is y = 0.29x − 14.77, R2 = 0.1, P = 0.03 (dashed line). Note that this equation is similar in slope and intercept to the linear regression relationship for juvenile birds (y = 0.385x − 14.67, R2 = 0.14, P = 0.07) (solid line)). (TIF) [file pone.0226318.s002.tif]

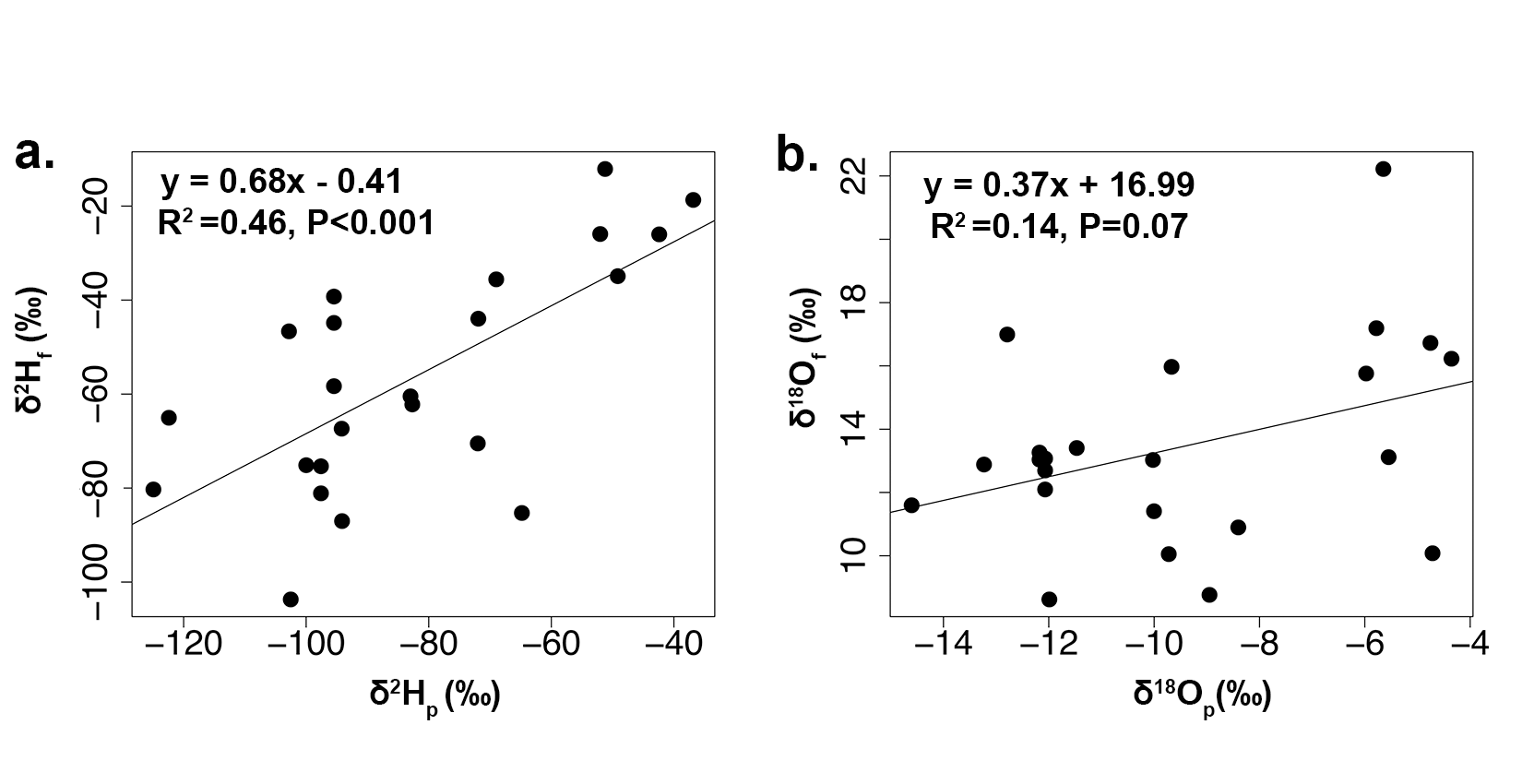

Supplement: S4 Fig — Stable hydrogen (δ2HF ‰) and oxygen (δ18OF ‰) isotopic composition of feathers for museum juvenile Sharp-shinned Hawk (Accipiter striatus) specimens (n = 23) of known natal origin and the isoscape modeled isotopic compositions of precipitation (δ2HP and δ18OP ‰) at the collection locations: (a) δ2HP values of birds versus δ2HF values, (b) δ18OP values of birds versus δ18OF values. (TIF) [file pone.0226318.s004.tif]

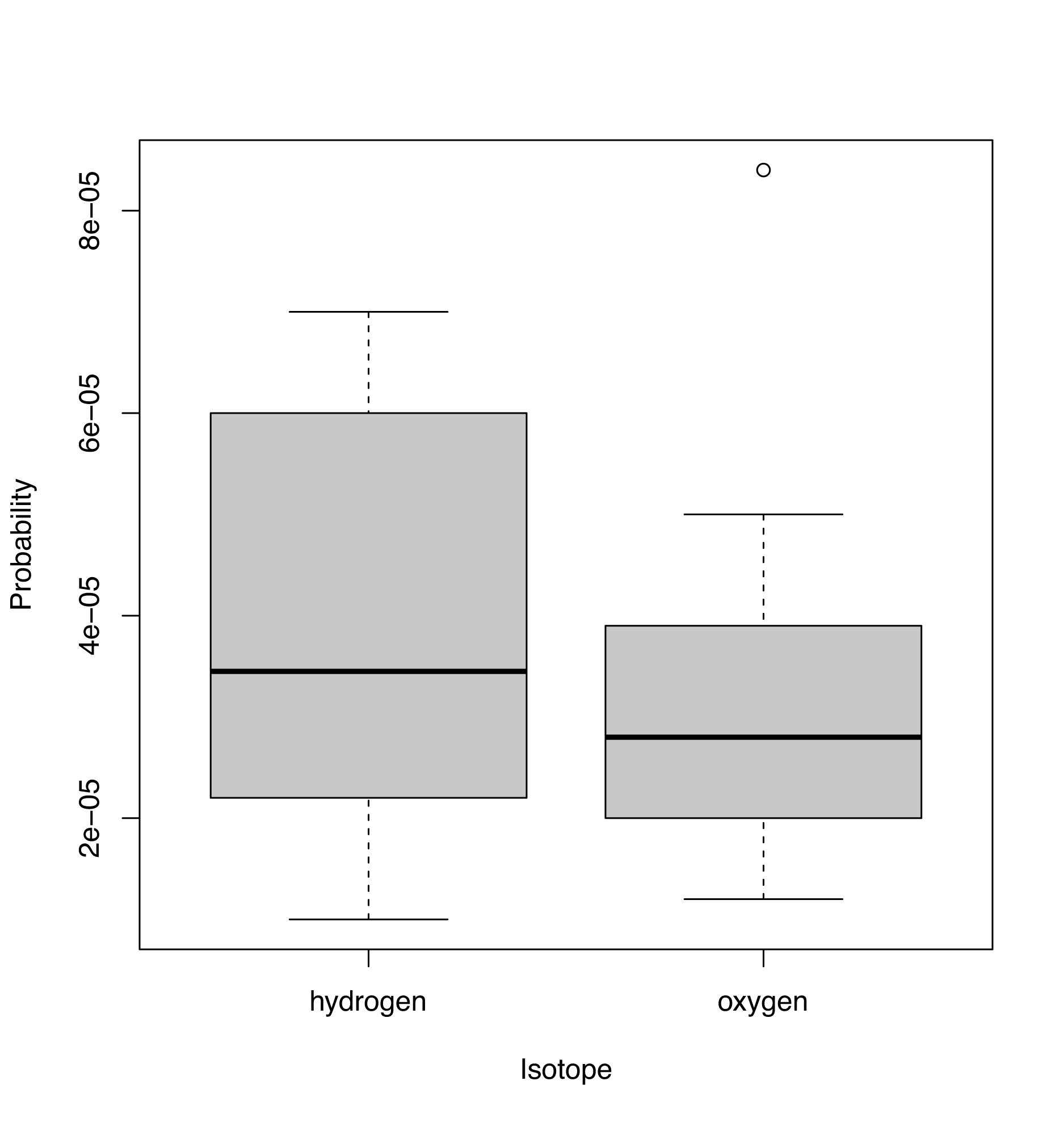

Supplement: S6 Fig — The boxplot compares relative probability values at the known collection site for museum specimens using transfer functions for δ2HP and δ18OP (n = 10). Probability density surfaces for each specimen were normalized so that values are comparable. The mean (± standard deviation) relative probability at the known collection sites was (3.98 ± 2.0) * 10−5 for hydrogen surfaces and (3.3 ± 2.1) * 10−5 for oxygen surfaces. (TIF) [file pone.0226318.s006.tif]

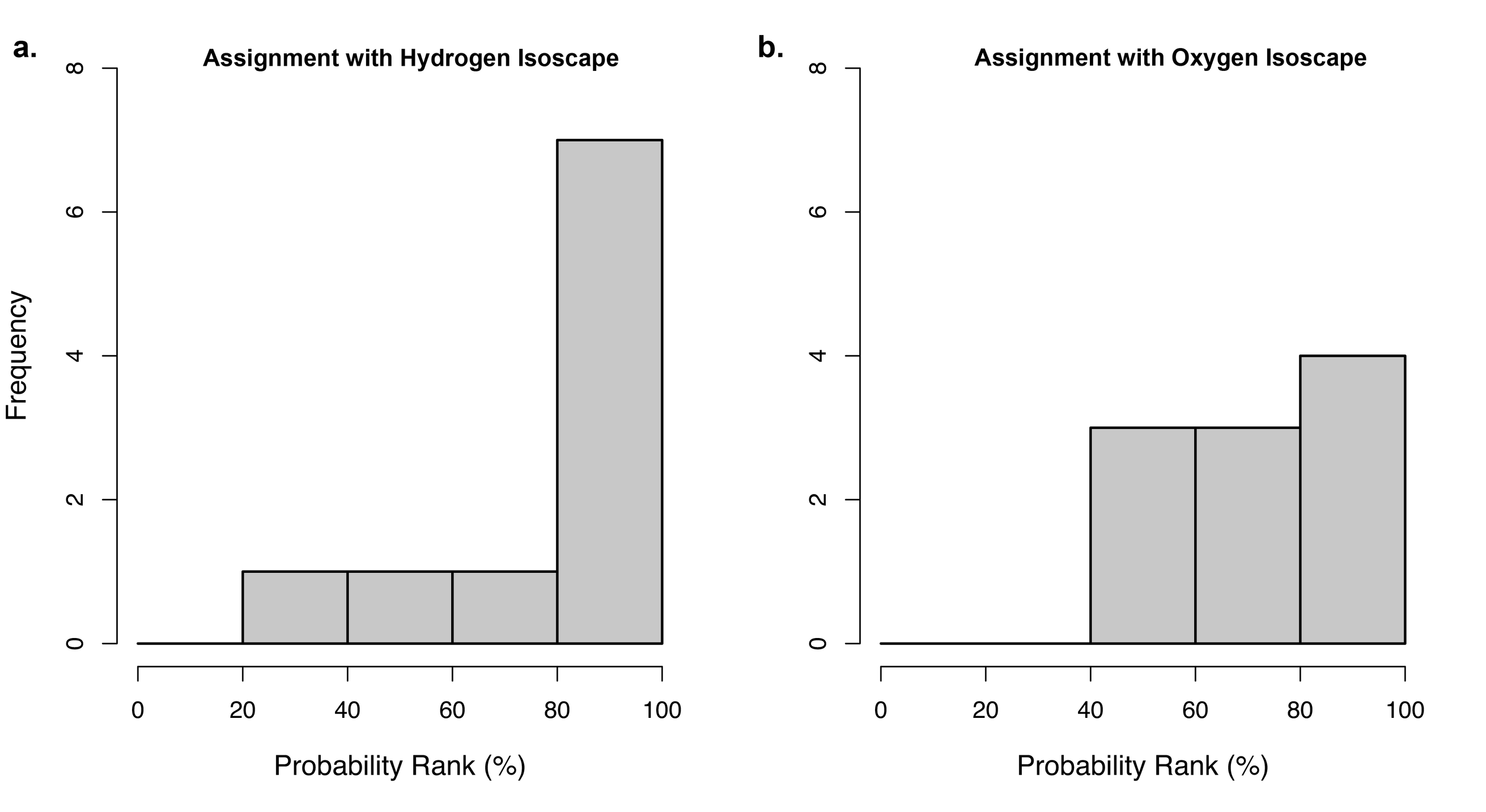

Supplement: S7 Fig — Histograms are based on (a) predicted δ2HP values (‰) and (b) predicted δ18OP values (‰) for birds captured at known locations (n = 10). Each value represents the relative probability that a sample of known origin was captured in a location that the isotope models predict as the origin. A value of 90–100% indicates the highest relative probability or that the isotope transfer function did a very good job of predicting the location that a sample was actually captured. A relative probability of 0–10% indicates a very poor match between the model prediction and actual capture site. (TIF) [file pone.0226318.s007.tif]

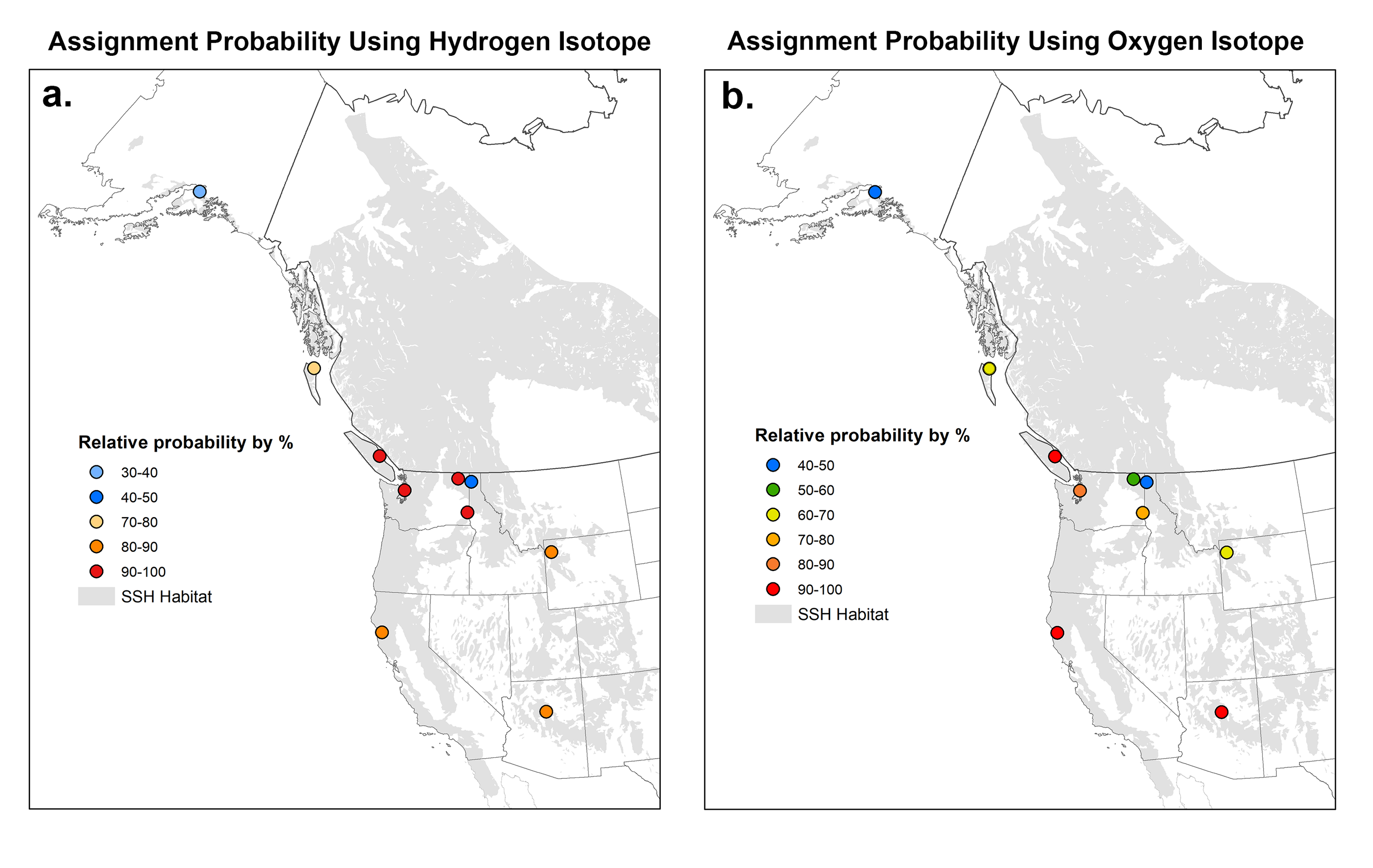

Supplement: S8 Fig — Relative probability is based on (a) predicted δ2HP values (‰) and (b) predicted δ18OP values (‰) for birds captured at known locations. Each point represents a specimen collection location. The color of the point represents the relative probability that a sample of known origin was captured in a location that the isotope models predict as the origin. A value of 90–100% indicates the highest probability or that the isotope transfer function did a very good job of predicting the location that a sample was actually captured. A relative probability rank of 0–10% indicates a very poor match between the model prediction and actual capture site. State and country boundaries are from public domain GIS files US Census Bureau (2016) and Natural Earth (2020). Species range acquired with permission from BirdLife International and NatureServe (2015), and data to create the GIS biome layer acquired with permission from Brown, Bennan, and Unmack (2007). (TIF) [file pone.0226318.s008.tif]
