# Supplementary material for: Using oxygen and hydrogen stable isotopes to track the migratory movement of Sharp-shinned Hawks (Accipiter striatus) along Western Flyways of North America
Source: PLoS One. 2020 Nov 17;15(11):e0226318. doi: 10.1371/journal.pone.0226318 (PMC7671529; doi:10.1371/journal.pone.0226318)
Supplement: S2 Table — Stable hydrogen (δ2HF ‰) and oxygen (δ18OF ‰) isotopic composition of feathers for juvenile museum Sharp-shinned Hawk (Accipiter striatus) specimens of known natal origin and estimated isotopic compositions of precipitation (δ2HP and δ18OP ‰) from the transfer functions used to determine assignment of origin. The states where samples were collected, and the museum Specimen IDs are included. (DOCX) [file pone.0226318.s010.docx]

**S2 Table: Stable isotopic compositions of museum feathers and estimates of precipitation isotopes used to test assignment of origin models.** Stable hydrogen (δ^2^H_F_ ‰) and oxygen (δ^18^O_F_ ‰) isotopic composition of feathers for juvenile museum Sharp-shinned Hawk (*Accipiter striatus*) specimens of known natal origin and estimated isotopic compositions of precipitation (δ^2^H_P_ and δ^18^O_P_ ‰) from the transfer functions used to determine assignment of origin. The states where samples were collected, and the museum Specimen IDs are included.

| **Hydrogen Transfer Function** | **^a^Museum Specimen ID** | **Measured δ^2^H_F_** | **Estimated δ^2^H_p_** | **^b^Locality** |
| --- | --- | --- | --- | --- |
| δ^2^H_p_=(δ^2^H_F_*0.68)-43.98, RSE 18.3 | MVZ 144621 | -46.7 | -75.7 | ID |
|  | UWYMV 1352 | -103.7 | -114.5 | WY |
|  | MVZ 169006 | -80.3 | -98.6 | AK |
|  | SDNHM 54234 | -26.0 | -61.63 | AZ |
|  | CRCM 89-223 | -67.4 | -89.81 | WA |
|  | CRCM 57-368 | -81.1 | -99.13 | WA |
|  | MVZ 81827 | -44.0 | -73.89 | BC |
|  | MVZ 99706 | -58.3 | -83.64 | BC |
|  | SDNHM 52830 | -35.6 | -68.20 | WA |
|  | MVZ 30835 | -12.1 | -52.22 | CA |

| **Oxygen Transfer Function** | **^a^Museum Sample ID** | **Measured δ^18^O_F_** | **Estimated δ^18^O_p_** | **^b^Locality** |
| --- | --- | --- | --- | --- |
| δ^18^O_p_=(δ^18^O_F_*0.385)-14.67, RSE 3.0 | MVZ 144621 | 16.99 | -8.13 | ID |
|  | UWYMV 1352 | 8.62 | -11.35 | WY |
|  | MVZ 169006 | 12.88 | -9.71 | AK |
|  | SDNHM 54234 | 17.19 | -8.05 | AZ |
|  | CRCM 89-223 | 11.41 | -10.28 | WA |
|  | CRCM 57-368 | 13.26 | -9.57 | WA |
|  | MVZ 81827 | 13.03 | -9.65 | BC |
|  | MVZ 99706 | 13.07 | -9.64 | BC |
|  | SDNHM 52830 | 10.90 | -10.47 | WA |
|  | MVZ 30835 | 22.22 | -6.12 | CA |

^a^Museums: CRCM = Charles R. Connor Museum; MVZ = Museum of Vertebrate Zoology; SDNHM = San Diego Natural History Museum; UWYMV = University of Wyoming Museum of Vertebrates

^b^States and Provinces: AK = Alaska, USA; AZ = Arizona, USA; BC = British Columbia, Canada; CA = California, USA; ID = Idaho, USA; WA = Washington, USA; WY = Wyoming, USA
