# Supplementary material for: Using oxygen and hydrogen stable isotopes to track the migratory movement of Sharp-shinned Hawks (Accipiter striatus) along Western Flyways of North America
Source: PLoS One. 2020 Nov 17;15(11):e0226318. doi: 10.1371/journal.pone.0226318 (PMC7671529; doi:10.1371/journal.pone.0226318)
Supplement: S1 Appendix — (DOCX) [file pone.0226318.s011.docx]

**S1 Appendix: Detailed information on the methods used to validate the test of the assignment of origin models for museum specimens of Sharp-shinned Hawks (*Accipiter striatus*) of known origin.**

We used samples of known origin from the museum samples to test the accuracy of the linear transfer functions and the assignment of origin models. We determined 10 known museum samples to test the Hydrogen isotope transfer function, selecting samples with the maximum values, the minimum values, the values closest to the mean (δ^2^H_F_ ‰), and samples that were from the geographic extremes of the area covered. We used the same 10 samples to also test the Oxygen isotope transfer function (Table S2). First precipitation isotope values were estimated for each feather value using the linear regression transfer functions that were developed previously (Figure 3). Then we generated individual probability surfaces for museum samples of known origin within the IsoMAP assignment function using the methods as described in the primary document (S5 Fig). Each map was normalized by calculating the sum of all grid values, then dividing each grid by that sum.

Once the validation maps were normalized, the relative probability at each specimen’s collection site was recorded. A boxplot was generated to compare the relative probability values at the site of known origin between Hydrogen and Oxygen isotopes (S6 Fig) Additionally, relative probability values were ranked by placing each grid value on one map into one of 10 “equal interval” categories in ArcGIS. The equal interval function divides the range of possible values on a map into 10 equal categories. The relative probability category was recorded for the capture location of each specimen such that the highest set of probability values were in the 90-100% category, the next set as 80-90% , the next as 70-80% and so on down to the lowest values 0-10%. Each value represents the relative probability that a sample of known origin was captured in a location that the isotope models predicted. A value of 90-100% indicates the highest probability or that the isotope transfer function does a very good job of predicting the location that a sample was actually captured. A relative probability rank of 0-10% indicates a very poor match between the model prediction and actual capture site.  The relative probability rank values were then plotted on histograms for each isotope (S7 Fig) and included on a map (S8 Fig). This allowed us to validate the calibration lines and interpret potential spatial patterns.
